# A comment on the Revised Diffusion Model for Conflict tasks (RDMC)

**DOI:** 10.3758/s13423-024-02574-5

**Published:** 2024-10-24

**Authors:** Markus Janczyk, Ian Grant Mackenzie, Valentin Koob

**Affiliations:** 1 Psychological Research Methods and Cognitive Psychology, Department of Psychology, University of Bremen, Hochschulring 18, D-28359 Bremen, Germany; 2 Eberhard Karls University of Tübingen, Tübingen, Germany

**Keywords:** Diffusion model, Diffusion Model for Conflict tasks, Parameter recovery, Inhibition, Decay

## Abstract

In conflict tasks, such as the Simon, Eriksen flanker, or Stroop task, a relevant and an irrelevant feature indicate the same or different responses in congruent and incongruent trials, respectively. The congruency effect refers to faster and less error-prone responses in congruent relative to incongruent trials. Distributional analyses reveal that the congruency effect in the Simon task becomes smaller with increasing RTs, reflected by a negative-going delta function. In contrast, for other tasks, the delta function is typically positive-going, meaning that congruency effects become larger with increasing RTs. The Diffusion Model for Conflict tasks (DMC; Ulrich et al., *Cognitive Psychology,* *78*, 148–174, 2015) accounts for this by explicitly modeling the information accumulated from the relevant and the irrelevant features and attributes negatively- versus positively-sloped delta functions to different peak times of a pulse-like activation resulting from the task-irrelevant feature. Because the underlying function implies negative drift rates, Lee and Sewell (*Psychonomic Bulletin & Review,* *31*(5), 1–31, 2024) recently questioned this assumption and suggested their Revised Diffusion Model for Conflict tasks (RDMC). We address three issues regarding RDMC compared to DMC: (1) The pulse-like function is not as implausible as Lee and Sewell suggest. (2) RDMC itself comes with a questionable assumption that different parameters are required for congruent and incongruent trials. (3) Moreover, we present data from a new parameter recovery study, suggesting that RDMC lacks acceptable recovery of several parameters (in particular compared to DMC). In this light, we discuss RDMC as not (yet) a revised version of DMC.

**Supplementary Information:**

The online version contains supplementary material available at 10.3758/s13423-024-02574-5.

How conflicting information is processed and affects performance is a highly investigated topic. Three prototypical tasks are mainly used in experimental research: the Simon task (Simon, 1969; Simon & Small, 1969), the Eriksen flanker task (Eriksen & Eriksen, 1974), and the Stroop task (Stroop, 1935). The common idea of these tasks is that a relevant and an irrelevant feature either indicate the same response or different responses in congruent and incongruent trials, respectively. The observation of slower and more error-prone responses in incongruent relative to congruent trials is the *congruency effect*. In addition, distributional analyses via so-called delta functions reveal that the size of the congruency effect changes across response times (RTs). These delta functions visualize the difference between congruent and incongruent RT quantiles against their mean (e.g., De Jong et al., 1994; Speckman et al., 2008). In the (visual horizontal) Simon task, the congruency effect typically becomes smaller with increasing RTs, reflected by a negatively-sloped delta function. In other tasks, like the flanker or Stroop task, the congruency effect usually becomes larger with increasing RTs, reflected by a positively-sloped delta function.

The congruency effect is often explained by the interplay of a controlled and an automatic channel in dual-route models (see, e.g., Kornblum et al., 1990). In congruent trials, both channels gather activation for the same response, while in incongruent trials, they gather activation for different responses.

**Fig. 1:**
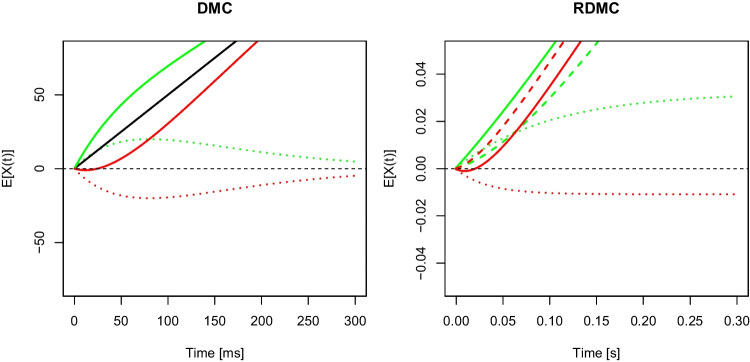
*Left panel*: DMC (Ulrich et al., 2015), *right panel*: RDMC (Lee & Sewell, 2024). *Note*. For DMC, the *solid black line* represents the expected time-course of activation within the controlled channel, the *dotted green* and *red lines *are the respective activations within the automatic channel, and the *solid green* and *red lines* are the overall expected activations from superimposing both channels (*green*: congruent, *red*: incongruent). For RDMC, the *dashed* and the* dotted lines* are expected activations within the controlled and the automatic channel, respectively. The *solid green *and* red lines* are the overall expected activations from superimposing both channels (*green*: congruent, *red*: incongruent). The figures were drawn with the following parameter values, DMC: $A=20$, $\tau = 80$, $a = 2$, $\mu _c = 0.5$; RDMC: $A_0=0.8$, $k_c=10$, $k_i = 30$, $d_c=0.6$, $d_a=0.4$ (see Table 3 and Figure 7 in Lee & Sewell, 2024). DMC is presented in milliseconds and RDMC in seconds for consistency with the original publications

Diffusion models developed for the case of conflict tasks also build upon this dual-route framework. These stochastic models allow one to disentangle the time-course and amount of evidence accumulated via the controlled and automatic channel. The *Diffusion Model for Conflict tasks* (DMC; Ulrich et al., 2015) is one such model that describes evidence accumulation within both channels as separate diffusion processes that are superimposed to yield a net activation (see also Hübner et al., 2010; White et al., 2011, for other diffusion models in the context of conflict tasks). Central to the model is that negatively versus positively sloped delta functions are attributed to different peak latencies of a pulse-like activation within the automatic channel that results from the task-irrelevant feature. Recently, this pulse-like activation pattern has been criticized as implausible and a *Revised DMC* (RDMC; Lee & Sewell, 2024) was proposed.

The present paper aims to discuss the critique of DMC, and subsequently offers a counter-critique of RDMC. Specifically, we argue that (a) the critique is not convincing and (b) RDMC comes itself with a questionable assumption. We then consider statistical properties and the parameter recovery properties of both RDMC and DMC. In conclusion, we highlight that RDMC provides a good fit to data from conflict tasks. However, we do not see that RDMC is (yet) a revised version or an advancement of DMC. We assume that readers are familiar with both models’ mathematics and will introduce them only briefly in the next section. More details are, however, provided in the Electronic Supplement A.

## Two models of conflict tasks

The main ideas of DMC and RDMC are visualized in Fig. 1 and will be briefly described in the next paragraphs.

### The Diffusion Model for Conflict tasks (DMC; Ulrich et al., 2015)

DMC builds upon the dual-route framework by describing the expected time-course of two evidence accumulation processes leading to response activations within the controlled and the automatic channel (see Fig. 1, left panel). These two activations are superimposed to yield the net activity of response activation which is used to select a response. The controlled route is based on a time-independent accumulation of evidence, similar to standard diffusion models (e.g., Ratcliff, 1978). Thus, the expected time-course of activation within the controlled route is a straight line with slope $\mu _c$ (the solid black line in Fig. 1). Since the expected time-course of activation in the controlled channel has a linear increase, $\mu _c$ is the first derivative with respect to time *t* and thus the drift rate of the controlled channel. The expected time-course within the automatic channel follows a pulse-like function that first increases and then decreases until reaching 0 again, and this "function represents the time-course of the expected mean of $\textbf{X}_a(t)$" (Ulrich et al., 2015, p. 168), that is, of the (response) activation within the automatic channel. DMC itself is agnostic to whether the decrease of activation after reaching a peak results from active inhibition (see Ridderinkhof, 2002) or mere passive decay (see, e.g., Hommel, 1994) (or both) of the activation that originally resulted from the irrelevant stimulus feature.

Technically, a rescaled Gamma function is used to describe the expected time-course of the automatic activation. The peak latency of this Gamma function is reached at $(a-1)\cdot \tau$, with *a* and $\tau$ being the shape and rate parameter, respectively. In addition, the Gamma function is multiplied with *A* to determine the amplitude (see the dotted green and red lines in Fig. 1, left panel). The direction into which the automatic activation is gathered is determined by the sign of *A*: In congruent trials, *A* has a positive sign, whereas it has a negative sign in incongruent trials. Thus, the activation in congruent and incongruent trials evolves in the same or opposite direction as the activation in the controlled channel. Similar to the case of the controlled activation, the respective drift rate is the first derivative of the Gamma function with respect to time *t* (see, e.g., Cox & Miller, 1965; Schwarz, 2022). Because the evidence added within the automatic channel in each time-step changes over time, the resulting drift rate $\mu _a(t)$ is time-dependent.

Superimposing both diffusion processes by adding them at each time-step yields the expected superimposed time-courses of activation in congruent and incongruent trials (see the solid green and red lines in Fig. 1, left panel). Whether a response is correct or incorrect, and its associated RT, is based on the superimposed process. In essence, DMC thus describes the assumed expected time-courses of (response) activation within the controlled and the automatic channel plus the combined activation resulting from their superimposition.

Because the absolute value of the expected activation in the automatic channel decreases after reaching its maximum, the respective drift rate $\mu _a(t)$ necessarily changes sign after this point. For example, $\mu _a(t)$ is first positive and then becomes negative in congruent trials (and vice versa in incongruent trials). In DMC’s framework this, however, does not necessarily mean that evidence for the other response is gathered after the peak, as the Gamma function only approaches zero with increasing time *t*. In other words, the decrease in (absolute) response activation, may it be negative or positive, merely reflects that evidence for one or the other response within the automatic channel decreases until eventually only the controlled channel continues to feed into the superimposed evidence accumulation process.

### The Revised Diffusion Model for Conflict tasks (RDMC; Lee & Sewell, 2024)

RDMC also builds upon the dual-route framework. In contrast to DMC, however, the basic idea is that both the controlled and the automatic channel continuously provide the same (rate of) information, but their relative influence changes over time. Whereas the contribution of the automatic channel is large at the outset of the diffusion process, it decreases with increasing time, and the contribution of the controlled channel increases reciprocally.

More precisely, an exponential decay function $w_a(t)$ models the decreasing contribution of the automatic channel, and that of the controlled channel is calculated as $w_c(t) = 1 - w_a(t)$. Multiplication of these weights with the base drift rate of both channels, $d_c$ and $d_a$, yields the time-dependent drift rates of the two channels, $v_c(t)$ and $v_a(t)$ (see the dotted and the dashed green and red lines in Fig. 1, right panel, for the expected values of evidence/activation). Because the exponential decay function is always positive, the expected value of activation within the automatic channel increases monotonically towards an asymptote. While the base drift rate for the controlled channel, $d_c$, is always positive, the base drift rate for the automatic channel, $d_a$, is positive on congruent and negative on incongruent trials.^1^

An important feature – one we will address in the next section – is that RDMC assumes separate decay rates of the exponential function (referred to as attentional shift parameters later) for congruent and incongruent trials. Thus, the asymptotic behavior of the automatic channel and the steepness of the increase in activation of the controlled channel are different in congruent and incongruent trials (see again the dotted and dashed lines in Fig. 1, right panel).

As in DMC, the superimposed drift rate at time *t* is derived by adding up the drift rates of both channels. Thus, it is also time-dependent and it differs for congruent and incongruent trials (see the solid green and red lines in Fig. 1, right panel, for the expected activations).

### A conceptual comparison

Although both DMC and RDMC are based on dual-route frameworks, they are conceptually different. DMC describes the theorized time-courses of the expected activation as a linear function and a pulse-like function for the controlled and the automatic channel, respectively. The corresponding drift rates of the respective evidence accumulation processes are then derived as the first derivative with respect to time. As a consequence, the drift rate within the automatic channel, $\mu _a(t)$, is time-dependent and changes sign in the course of a trial, while the drift rate within the controlled channel, $\mu _c$, remains constant (i.e., is time-independent).

RDMC, in contrast, assumes constant rates of evidence accumulation within the controlled and the automatic channel. However, both channels’ contribution changes across time in a reciprocal way. Thus, the contribution of the automatic channel decreases, and that of the controlled channel increases. Eventually, evidence accumulation is driven only by the controlled channel.

## Thoughts about assumptions

This section fulfills two purposes. We will first address the main critique of DMC raised by Lee and Sewell (2024), namely that the pulse-like function of activation in the automatic channel is implausible. We then turn to RDMC’s assumption of separate attention shift parameters for congruent and incongruent trials (i.e., the parameters that control how quickly the influence of the automatic channel decays).

### Inhibition, suppression, and the pulse-like activation of DMC

#### The argument

The main critique of Lee and Sewell (2024) of DMC concerns the "use of a pulse function to describe the cumulative output of the automatic channel [...]" which "enforces an evidence accumulation process involving rapid initial accrual of evidence based on distractor processing followed by the gradual withdrawal of that evidence from the decision process" (p. 6), and requires "selectively accumulating ’counter-evidence’ that is contrary to the properties of the distractor stimuli" (Lee & Sewell, 2024, p. 7). A similar reservation is conveyed by Heuer et al. (2023) who note that "the assumption that in the course of a rapid decision an inhibitory instantaneous influence of an irrelevant stimulus feature is inverted to become facilitating (and vice versa) lacks plausibility" (p. 4).

#### Counterarguments

We treat this argument in two steps. First, we address the plausibility of the pulse-like time-course of (expected) activation within the automatic channel. Second, we discuss its implications for the resulting time-dependent drift rate.

##### The pulse-like time-course of activation

DMC itself is agnostic as to whether the reduction of activation in the automatic channel is due to mere passive decay or an inhibitory mechanism acting on and reducing the activation, as suggested by Ridderinkhof (2002; see also Mittelstädt et al., 2023; Ridderinkhof et al., 2004). A possible implication of inhibition is also acknowledged by Lee and Sewell (2024, Footnote 5). Importantly, however, they "agree with the idea that inhibition can halt subsequent processing", that is, that inhibition can prevent further accumulation within the automatic channel (see also Logan, 1980; Logan & Zbrodof, 1979), but they are doubtful "that inhibition can reverse or undo the effects of previously completed processing" (p. 6). The first aspect of the argument is thus about whether one believes that activation within the automatic channel, once gathered, (1) must remain in the system (the view noted by Lee & Sewell, 2024) or (2) can decay or be reduced as well (as assumed by DMC; Ulrich et al., 2015). We believe that there are good reasons for the latter view.

We will not go into the issue of passive decay here further, as we hardly can conceive a cognitive system that never loses gathered activation again (see Ulrich et al., 2015, for arguments in favor of decay), but rather showcase examples where inhibition is proposed as a means to reduce activation. Perhaps the most well-known mechanism of this kind is lateral inhibition, a concept that "refers to the capacity of excited neurons to reduce the activity of their neighbors" (Cohen, 2011, p. 1436). Here, we neither want to deal with inhibition on the neural level nor with its counterpart in connectionist network models (where, according to our impression, not much dissent exists that inhibition reduces activation; see also MacLeod, 2007). Rather, we outline several – and necessarily selected – examples that conceive inhibition as a cognitive process aiming at a reduction of activation:

- *Task Switching.* The first example comes from task-switching, that is, when participants have to deal with varying tasks on a series of trials. A typical result is the observation of switch costs: If the current task on trial *n* is different from the task on the preceding trial $n-1$, RTs become longer (and more errors are made). These switch costs have been attributed to several underlying causes, including preparatory processes thought to (re-)activate and implement the currently relevant task set (see Kiesel & Koch, 2022; Kiesel et al., 2010; Koch et al., 2010, for reviews). In a seminal paper, Mayr and Keele (2000) argued, however, that another, inhibitory, element is required: "Selecting against a just-executed set could be accomplished by deactivating it, thus, providing the necessary ’room to move’ for selecting the new task set." (p. 5). Crucially, what the authors termed *backward inhibition* is explicitly conceptualized as *deactivating* the previous task set, thus reducing its activation to facilitate the selection of the now required one. Evidence for this proposal was gathered from what has later been termed $n-2$ repetition costs: Imagine, participants work on and switch between three tasks, A, B, and C. The critical comparison is between sequences such as ABA and sequences such as CBA. A mere sustained activation account predicts shorter RTs to the final task A in ABA than in CBA sequences, as task A has recently been encountered and should thus be highly accessible (though some passive decay might have occurred, of course). In contrast, the backward inhibition account predicts the opposite. Switching away from task A implies its inhibition. When A is required soon again in ABA sequences, it has recovered less from this inhibition than in CBA sequences. As a consequence, longer RTs are predicted in ABA than in CBA sequences. In fact, such $n-2$ repetition costs have been obtained many times. Because other accounts (such as one based on expectancy) seem unlikely, explaining $n-2$ repetition costs in terms of inhibition is regarded as compelling (for reviews, see Koch et al., 2010; Mayr, 2007). While some issues are subject to debate (e.g., whether recovering from backward inhibition is time- or event-based; see Gade & Koch, 2005), it seems clear that $n-2$ repetition costs can be explained only when construing backward inhibition as a process that actively reduces the activation of a task set, rather than only preventing activation from a further increase.
- *Attentional Capture.* A second example comes from the visual search and attentional capture literature. Put simply, when an observer searches for a defined target in a search array, salient distractors can (involuntarily) capture attention, thereby interfering with the search task. Two opposing camps suggested either that salient stimuli will attract attention (Theeuwes, 1991, 1992, 2023) or that only stimuli matching the observer’s intention can do so (“contingent capture”; Folk et al., 1992; Luck et al., 2021). To reconcile these theoretical positions, Sawaki and Luck (2013) proposed the signal suppression hypothesis, an idea similar to the one introduced by Ridderinkhof (2002) in the context of conflict tasks (see above). The basic idea is that salient stimuli can yield sufficient activation to attract attention. Yet, a subsequent inhibitory process can be applied to reduce this activation, thereby preventing attentional capture. There is a large literature providing empirical evidence for this idea, including a specific ERP component, the distractor positivity (Pd), thought to index suppression of an item (for reviews, see Gaspelin & Luck, 2018; Gaspelin et al., 2023). Important for the present purposes is evidence that processing at particular locations can be “inhibited below baseline levels of processing” (Gaspelin & Luck, 2018, p. 86). Conceivably, this can only be achieved if the inhibitory process is able to actively reduce a salience or priority signal such that allocation of attention to this location becomes less likely (and as a consequence, items at this location are less processed). In a similar direction, Liesefeld and Müller (2021; see also Moran et al., 2013) conceptualized item selection by means of evaluating the item’s activation relative to all other items’ activation (see also Luce, 1986, for this selection rule). For example, suppose the selected item turns out as not being the target, its activation is suppressed (see Liesefeld & Müller, 2021, p. 723) to ensure that the same item is not selected over and over again.
- *Memory.* A third example comes from memory research, and perhaps most prominently as an explanation for retrieval-induced forgetting (RIF; Anderson et al., 1994). Retrieving a subpart of initially learned items facilitates their subsequent availability in recall or recognition tests. RIF is the observation that this retrieval also comes with a disadvantage: Items related to the retrieved items (e.g., by sharing category membership) are later *less* accessible than other, but unrelated items. This result has been replicated in many studies (see Bäuml et al., 2010; Murayama et al., 2014; Verde, 2012, for reviews and a meta-analysis). A prominent account for RIF is based on inhibition. More precisely, the suggestion is that "inhibition may act during retrieval to suppress, or diminish the accessibility of, interfering items, to facilitate the retrieval of target items" (Storm & Levy, 2012, p. 828). In this sense, "inhibition is a functional mechanism that acts with the specific and direct purpose of reducing the accessibility of an item or items in memory" (p. 829). One of the main arguments for this view is that items related to the retrieved items are also remembered worse, when a different retrieval cue is presented at test ("cue-independent forgetting"; see also Anderson, 2003). Hence, "inhibition acts to decrease the activation of nontarget items directly" (Storm & Levy, 2012, p. 831). Of course, other non-inhibitory accounts have been proposed (see Jonker et al., 2013; Raaijmakers & Jakab, 2013; Verde, 2012). However, the inhibition account still seems to be the dominating one (see Storm & Levy, 2012, for a review).

These three examples – from several fields of psychological research – illustrate that inhibition is often seen as a means to reduce activation. Accordingly, inhibition may well be seen as a plausible cause of the reduction of (response) activation (see Ridderinkhof, 2002), which yields the pulse-like time-course of activation described in DMC. Note also that López and Pomi (2024) provided a model where inhibition of activation is explicitly modeled as being dependent on the activation within the controlled channel. Their model does not a-priori assume a particular form of the time-course of the expected activation within the automatic channel, but a pulse-like function emerges from the model dynamics.

##### The implied time-dependent drift rate of the automatic process

Above, we have argued that the pulse-like form of the expected activation within the automatic channel is plausible. As mentioned in the introduction, deriving the drift rate as the first derivative with respect to time *t* results in a time-dependent drift rate $\mu _a(t)$ that changes sign in the course of a trial. According to Lee and Sewell (2024) this indicates "selectively accumulating ’counter-evidence’ that is contrary to the properties of the distractor stimuli" (p. 7; see also Heuer et al., 2023). We believe that the potential disagreement here derives from two different interpretations of the drift rate.

First, Ulrich et al. (2015) adopt a technical interpretation focusing on the mean change of the activation *X*(*t*) at time *t* (Cox & Miller, 1965; Schwarz, 2022). In this view, the changing sign of the drift rate simply is the necessary result of the pulse-like activation within the automatic channel (the form of which, we have argued above, is plausible in our opinion).^2^ Additionally, the sign must not be interpreted as indicating evidence accumulation for one or the other response in this case: The (expected) activation within the automatic channel is always either for one *or* the other response; only the amount of the activation present within the channel first increases and then fades to zero.

Second, Lee and Sewell (2024) seem to adopt an interpretation that links drift rates to stimulus quality (see, e.g., Ratcliff & McKoon, 2008), as it is common for time-independent drift rates. In this case, a positive drift rate indeed indicates evidence for the response associated with the upper boundary, while a negative drift rate indicates evidence for the response associated with the lower boundary.

#### Evaluation

The preceding paragraphs dealt with the main critique raised by Lee and Sewell (2024) against DMC, namely that the assumption of a pulse-like form of the (expected) activation within the automatic channel is implausible.

First, we showcased examples from various fields of cognitive psychology research where – to our understanding – inhibition refers to a process that (actively) reduces activation (of various kinds).^3^ As a consequence, we conclude that the pulse function assumed by DMC is plausible (see also López & Pomi, 2024). Second, we argue that the sign-change of $\mu _a(t)$ is a natural by-product when understanding the drift rate as the first derivative of the expected activation with respect to time *t*. This reflects that response activation within the automatic channel starts to decrease back toward zero.

Sure, our opinion is not carved in stone; yet, we are not convinced that the main criticism of DMC applies. In the next section, we consider an assumption of RDMC that we find implausible.

### Congruency as a high-order emergent feature

#### The argument

The core idea behind RDMC is that attention is shifted away from the automatic channel with increasing time thereby reducing the impact of irrelevant features. Critically, RDMC allows "the attention shift parameter, *k*, to differ for congruent and incongruent trials" (Lee & Sewell, 2024, p. 9), giving rise to the two parameters $k_c$ and $k_i$ for congruent and incongruent trials.^4^

This raises the question: How does the cognitive system know at the onset of the decision process whether the trial is congruent or incongruent? Lee and Sewell (2024) argue that congruency is "a high-order emergent feature of the stimulus configuration that is based on the homogeneity of stimulus elements (e.g., as in flanker tasks) or the conceptual alignment of elements (e.g., as in Stroop and Simon tasks)" (p. 9). Several problems we see for this assumption are outlined in the next paragraph.

#### Counterarguments

Let us first consider the flanker task, of which many different versions exist. If one uses the arrow variant, where the central arrow determines the response and is flanked by arrows to its left and right, the perceptual impression is indeed homogeneous in congruent trials (all arrows point to the same direction; < < <) but not in incongruent trials (target and flanker arrows point to different directions; < > <). The same might be true in versions where, for example, two letters are used as targets and flankers (e.g., HHH vs. HSH).

These situations are, however, not the only possibilities. Already Eriksen and Eriksen (1974) were aware that mapping more stimuli to single responses is necessary to isolate response conflict from perceptual homogeneity. For example, suppose that the letters H and X require a left response and S and O require a right response. The stimulus configuration HXH is then response congruent, but the perceptual impression is certainly not homogeneous. Studies on the flanker task often use this or similar versions (e.g., Miller, 1987; Verbruggen et al., 2006; Wühr & Heuer, 2017).^5^

The situation is even less compelling for typical Simon tasks, where, for example, a letter or color determines the left or right response location while the stimulus itself is presented to the left or right. Intuitively, one needs some information about the required response to determine whether it is congruent or not with the stimulus location. Since extraction of this information is exactly what the diffusion process is about, it is difficult to conceive how this information already affects the beginning of the diffusion process.^6^

Lee and Sewell (2024) seem aware of these problems and decouple the (preceding) identification of congruency from the response selection (i.e., the diffusion) process. They assume "that the detection of conflict does not rest on knowledge of the target" (p. 9) and suggest a "conflict detection mechanism" (p. 9) that identifies conflict on the basis of activation resulting from stimulus properties. Although possible, one might note though that conflict measurement (or more precisely: energy measurement) is implemented in the *response* layer for Stroop and flanker tasks in the conflict monitoring model by Botvinick et al. (2001).

One might avoid the a-priori conflict detection mechanism by speculating that participants learn the congruency level of a target/distractor combination on the first few trials of an experiment, so that congruency information can be rapidly inferred merely on the percept of the stimulus configuration prior to response selection. We concur that this might be a feasible solution in defense of the two attention shift parameters $k_c$ and $k_i$. Yet, it at least makes the model much less parsimonious than DMC.

A further question is (if we put the learning account aside for the moment): If some activation from the different stimulus properties gains entry into the cognitive system, what happens with this activation (in particular with that resulting from the response-determining stimulus properties)? As far as we understand, this information is not further used in the subsequent progress of the diffusion process. There are instances, where flushing of activation seems appropriate in dual-tasking (see Koob, Ulrich et al., 2023; Logan & Gordon, 2001), but it is unclear whether this also makes sense in a single-task like those being dealt with here.

There are additional puzzling pieces. RDMC assumes different base drift rates $d_a$ and $d_c$ for the automatic and the controlled channel. According to Lee and Sewell (2024, p. 11) the

...channel-specific base drift rates describe the maximum levels of stimulus quality provided by task-irrelevant and task-relevant components of the stimulus (i.e., they index latent discriminability when attention is focused exclusively on either target or distractor information).

Arguably, processing the left/right location of a stimulus is faster than identifying the stimulus identity in Simon tasks (see, e.g., Hilchey et al., 2011; Mittelstädt et al., 2023). Yet, when applying RDMC to Simon data (taken from Ulrich et al., 2015, see also below), $d_a$ was smaller than $d_c$ (Table 4 in Lee & Sewell, 2024). Additionally, Lee and Sewell (2024) conclude "that negative-going delta plots, like those observed in the Simon task, arise when distractor information is not rapidly filtered out on congruent trials." (p. 19). In other words, negative-going delta functions arise when the decision is heavily based on the distractor information in congruent trials but on the target information in incongruent trials. This makes sense, as the distractor is beneficial in congruent trials, but harmful in incongruent trials. Yet, because RDMC estimates $d_a$ to be smaller than $d_c$ in the Simon task, this then brings up the question of why a participant relies heavily on the task-irrelevant stimulus in congruent trials if it has a lower quality than the task-relevant stimulus feature.

Furthermore, if a cognitive system knows that the current trial is congruent, even before the actual response decision starts, why should it not rely entirely on the distractor information? Indeed, there is evidence from congruency pre-cueing studies that putative different processing in congruent and incongruent trials only reflected a strategy shift toward responding to the location information when the pre-cue signals a congruent trial (Wühr & Kunde, 2008).

#### Evaluation

One critical assumption of RDMC is that the attention shift parameter differs between congruent and incongruent trials. Logically, this requires the cognitive system to know the congruency status already at the beginning of the response selection process, that is, of the diffusion process. We acknowledge that Lee and Sewell (2024) offer a solution to this by assuming an additional "conflict detection mechanism" (p. 9) that – logically – must operate already on the same stimulus information prior to the response selection process proper. Adding this extra complexity might, however, not be desired for the sake of parsimony. In addition, it is unclear why this congruency information prior to response selection is not used to implement an efficient decision process (at least in the Simon task).

### Summary

The preceding paragraphs first addressed Lee and Sewell ’s (2024) critique of the pulse-like function assumed by DMC (Ulrich et al., 2015) for the activation within the automatic channel. We conclude that this particular form is plausible, yet different interpretations of the drift rate might be entertained. We secondly discussed RDMC’s requirement of separate attention shift parameters for congruent and incongruent trials. We acknowledge that Lee and Sewell (2024) suggest a possible mechanism for determining the congruency status even before the diffusion process starts. Yet, we do not find the grounding in perceptual "homogeneity of stimulus elements" (p. 9) for many standard stimulus configurations convincing. In addition, the added mechanism adds complexity and renders RDMC less parsimonious.

Given this state, we, so far, cannot conclude that RDMC is a more theoretically compelling revised version of DMC. We continue by addressing the statistical and measurement properties of both models.

## A statistical comparison of DMC and RDMC

Since both DMC and RDMC can fit the data of the Simon and flanker task well, a qualitative and formal comparison can help elucidate the costs and benefits of each model. We used the Simon and flanker data of Ulrich et al. (2015) in the following, as these data were also used in the original publications on DMC (Ulrich et al., 2015) and RDMC (Lee & Sewell, 2024). Both the Simon and flanker dataset comprise 336 trials (168 per compatibility condition), obtained from the same 16 participants who block-wise alternated between the tasks in a single session.

As formulated in the original publication, RDMC comprises five parameters related to target and distractor processing (i.e., $A_0$, $k_c$, $k_i$, $d_c$, $d_a$), while DMC typically comprises 3 parameters (i.e. *A*, $\tau$, and $\mu _c$; note that we fixed the shape parameter of the automatic process to $a = 2$, as in Ulrich et al., 2015). Additionally, both models employ a parameter for the decision boundary *b* and two parameters for the residual process (often called the non-decision time; $t_0$, and $s_{t0}$). Although DMC was originally introduced with a normally distributed residual time, RDMC employs a uniform distribution (Ratcliff, 1978). Yet, for typical parameter ranges and configurations, both can be used interchangeably (see also Ratcliff, 2013). However, in the following modeling exercise, we used a uniform distribution for both DMC and RDMC to keep them as similar as possible. Then $t_0$ is the expected value of the uniform distribution and $s_{t0}$ indicates its range. In sum, RDMC thus employs eight parameters ($A_0$, $k_c$, $k_i$, $d_c$, $d_a$, *b*, $t_0$, and $s_{t0}$) and DMC employs six parameters (*A*, $\tau$, $\mu _c$, *b*, $t_0$, and $s_{t0}$). We first present results from a formal model comparison taking into account the different numbers of parameters and then turn to measurement properties in a parameter recovery study.

Both DMC and RDMC were estimated via maximum likelihood as implemented in the R package dRiftDM (Koob et al., 2024, version 0.1.1). Model predictions (i.e., probability density functions) were derived in the unit of seconds and with a diffusion constant of $\sigma = 1$ by numerically approximating the Kolmogorov forward equation via Crank-Nicolson (see Richter et al., 2023, for more information). Note that RDMC was originally introduced with a diffusion constant of $\sigma = 0.1$ and in the unit of seconds. DMC, in contrast, was introduced with a diffusion constant of $\sigma = 4$ and in the unit of milliseconds. Thus, the scaling of parameters in the present study is different from the scaling in the original articles. We provide equations on how to transform parameters in the Electronic Supplement B. The step size for discretizing the time and evidence space was set to $\Delta x = \Delta t = 0.002$. Differential evolution (Storn & Price, 1997) was used for minimizing the negative log-likelihood with the R package DEoptim (Ardia et al., 2011). The parameter space used when estimating the models is shown in Table B1 (see Electronic Supplement B). Note that, unlike a SIMPLEX algorithm (Nelder & Mead, 1965), Differential Evolution does not rely on providing starting values. Rather, lower and upper bounds are provided spanning the complete parameter space.

**Fig. 2:**
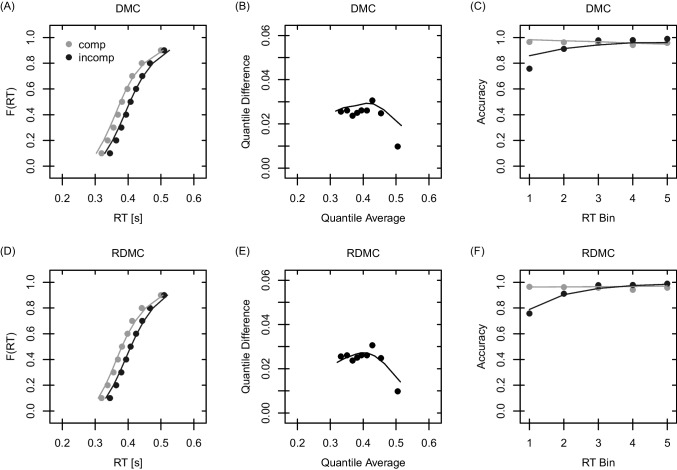
DMC (*upper row*) and RDMC (*lower row*) predictions for the Simon task data. *Note*. In all panels, model predictions are shown as *lines*, and observed values are shown as *dots*. Specific values were obtained by averaging across participants. The leftmost panels (i.e., **A** and **D**) show predicted and observed quantiles. The middle panels (i.e., **B** and **E**) show delta functions which can be derived by plotting the difference between quantiles (to the same probability) of incompatible and compatible trials against their mean. The rightmost panels (i.e., **C** and **F**) show CAFs, derived by first binning RTs and then calculating the proportion of correct responses per bin

### Model fit and information criteria

We first present our results when fitting both DMC and RDMC to the data of Ulrich et al. (2015). However, an issue left open is the potential inclusion of trial-by-trial variability in the starting point when fitting observed data. When fitting DMC, it is often necessary to include such variability, for instance, in the shape of a Beta function (see Ulrich et al., 2015). The reason is that data from conflict tasks usually show a tendency toward fast errors in incompatible trials, and this tendency can only partially be accounted for by DMC otherwise. RDMC, however, does not require trial-by-trial variability in the starting point to model data with only mild tendencies for fast errors (for instance, when fitting the data of Ulrich et al., 2015; see Lee & Sewell, 2024, p. 11). Therefore, it is not clear how to make a fair comparison. Comparing both models without variability in the starting point is disadvantageous for DMC. In turn, introducing trial-by-trial variability in the starting point for RDMC makes it more complex than necessary. Since our focus in the present study is on task processing within the automatic and controlled channel, we decided to fit DMC without variability in the starting point.^7^ In the Electronic Supplement C, we present the results when additionally fitting trial-by-trial variability for DMC.

**Fig. 3:**
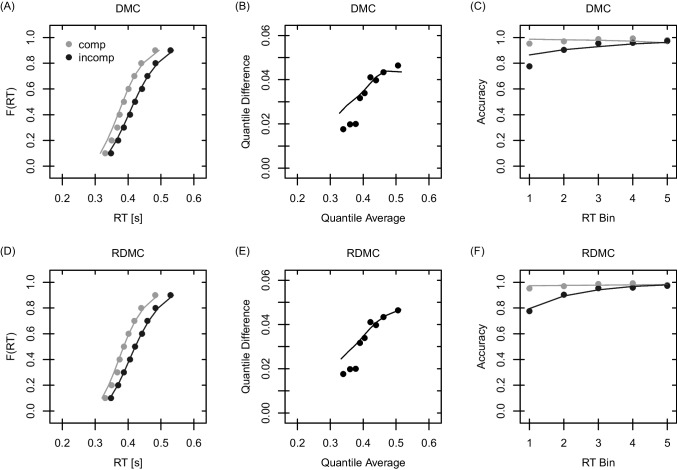
DMC (*upper row*) and RDMC (*lower row*) predictions for the flanker task data. *Note*. Figure legend is identical to Fig. 2

Qualitative model fits to the Simon and flanker data are visualized in Figs. 2 and 3, respectively. Each figure shows the observed and predicted quantiles, delta functions, and conditional accuracy functions (CAFs). Qualitatively, both DMC and RDMC fit the data sets well. For the Simon and flanker task, however, RDMC qualitatively outperforms DMC in predicting fast errors (see the first bin of the CAFs). In addition, RDMC outperforms DMC when predicting the congruency effect for slow responses in the Simon task (see the last quantile difference of the delta function).^8^

Next, we investigated for each individual participant whether DMC or RMDC performs best with respect to the plain log-likelihood, the Akaike Information Criterion (AIC), and the Bayesian Information Criterion (BIC). Table 1 summarizes the results by presenting the respective frequencies alongside the average log-likelihood, AIC, and BIC values (the latter were obtained by averaging across all participants, not only those for which a model turned out best). Given the search space as listed in Table B1, we observed RDMC to outperform DMC with respect to the plain log-likelihood. When correcting for the number of parameters, RDMC outperformed DMC with respect to the AIC statistic. When considering the more conservative BIC statistic, RDMC and DMC were comparable.

**Table 1:** Summary of log-likelihood, AIC, and BIC values for DMC and RDMC, separately for the Simon and flanker data sets

	Log-likelihood		AIC		BIC	
Dataset/Model	*M*	*N*	*M*	*N*	*M*	*N*
Simon						
DMC	353	2	$-695$	2	$-672$	10
RDMC	359	14	$-702$	14	$-672$	6
Flanker						
DMC	370	2	$-727$	7	$-705$	9
RDMC	375	14	$-734$	9	$-704$	7

AIC = Akaike Information Criterion, BIC = Bayesian Information Criterion, *M* = Mean, *N* = Number of participants for which a certain statistic was largest (log-likelihood) or smallest (AIC and BIC), that is, for which a certain model turned out ’best’

### Parameter recovery

In order for a model to be diagnostic with respect to the individual parameter estimates, its parameters must be estimable with at least some degree of precision. The simplest way to investigate this property is to conduct a parameter recovery study, where a model’s parameters are estimated based on synthetic data sets that are simulated from a set of randomly drawn (and thus known) original parameters. Ideally, the estimated and original parameters underlying the synthetic data sets match, and the degree to which they match can be expressed via the correlation coefficient.

**Table 2:** Correlations from the parameter recovery study

	RDMC						
	$A_0$	$k_c$	$k_i$	$d_a$	$d_c$	*b*	$t_0$
$N = 200$	0.38	0.22	0.35	0.55	0.88	0.94	0.99
$N = 500$	0.22	0.18	0.54	0.38	0.96	0.96	0.99
$N = 1000$	0.40	0.27	0.63	0.53	0.96	0.97	$>0.99$
$N = 10,000$	0.54	0.52	0.85	0.86	$> 0.99$	$>0.99$	$>0.99$
	DMC						
	$\mu _c$	*A*	$\tau$			*b*	$t_0$
$N = 200$	0.98	0.86	0.72			0.95	0.99
$N = 500$	0.99	0.93	0.86			0.98	$> 0.99$
$N = 1000$	$> 0.99$	0.98	0.91			0.99	$> 0.99$
$N = 10,000$	$> 0.99$	0.99	0.99			$>0.99$	$> 0.99$

Lee and Sewell (2024) report almost perfect parameter recovery for RDMC with correlations being at least 0.87 and very often even $> 0.95$ (based on 40 data sets with either 200, 500, or 1000 trials per compatibility condition). However, the authors allowed the minimization routine to repeatedly start close to the original/generating parameters. Specifically, each synthetic data set was fitted ten times, and the starting points for each minimization run were within $\pm 10\%$ of the original parameter values. The final parameter set was then the best-fitting parameter set from all 10 runs. We argue that this procedure likely overestimated the correlations between estimated and original parameters in contrast to a more realistic scenario, where starting values are not selected close to the original values.

We thus conducted a new parameter recovery study for RDMC (and DMC) across a complete parameter space (with $s_{t0}$ being fixed to 0.07, as in Lee & Sewell, 2024). One-hundred sets of original parameters were randomly drawn from uniform distributions with ranges listed in Table B1 (see Electronic Supplement B). For each parameter set, the cumulative distribution functions (CDFs) of RTs were derived (with $\Delta x = \Delta t = 0.001$ in this case), and based on the CDFs, the synthetic data sets were generated by means of inverse transform sampling. The trial numbers were either 200, 500, 1000, or 10,000 per compatibility condition. While the trial numbers of 200 and 500 reflect trial numbers common in experimental data sets, the trial numbers of 1000 and 10,000 aim at investigating recovery property in the limit. RDMC and DMC were then fitted to the synthetic data sets via Differential Evolution, using the same procedure as above. Note that the parameter space when estimating the model was slightly larger than the parameter space when generating the original values.

Table 2 summarizes the obtained correlations. In addition, we present scatter plots of estimated against original parameter values in Figs. B1 and B2 (see Electronic Supplement B). As can be seen, parameter recovery for DMC was reasonably good (see also White et al., 2018), in particular with increasing trial numbers. In contrast, several parameters were not accurately recovered in the case of RDMC. This, of course, contrasts with the original study by Lee and Sewell (2024), but we believe this version is the fairer treatment given that we did not provide the minimization algorithm with close-to-optimal starting values.^9^

## Conclusion

The present manuscript addressed and evaluated the recently proposed RDMC (Lee & Sewell, 2024) against DMC (Ulrich et al., 2015). Our main disagreement with Lee and Sewell is concerned with two assumptions:

First, the time-course of the expected activation in the automatic channel was criticized as being implausible by Lee and Sewell (2024) as it implies a sign-change of the corresponding drift rate, which is traditionally be taken to indicate evidence accumulation for the other response. We argue that inhibition as a means to reduce existing activation is a rather common idea in cognitive psychology. Hence, the decreasing activation after reaching the maximum could – if not being the result of mere passive decay – easily be accounted for by an inhibitory mechanism (Ridderinkhof, 2002). We do not have a preference for the exact reason of the reduction (i.e., inhibition vs. decay or even both). The major point of disagreement here seems to be different interpretations of the drift rate. While Lee and Sewell (2024) seem to entertain the "traditional" interpretation of the drift rate as reflecting stimulus quality, DMC interprets the drift rate in a more technical sense as the first derivative of the expected mean activation with respect to time *t* (see Cox & Miller, 1965; Schwarz, 2022). Hence, although certainly open to discussion, we see DMC’s pulse-like function as plausible and the sign-change of the drift rate justified.

Second, RDMC assumes one important parameter to differ for congruent and incongruent conditions. Albeit certainly possible, this is an assumption we do find at least questionable. At the very least, adding a mechanism that acts before response selection to detect the congruency status introduces another complexity to RDMC. In addition, we believe that identifying congruency on the basis of "homogeneity of stimulus elements [...] or the conceptual alignment of elements" (Lee & Sewell, 2024, p. 9) limits RDMC to specific variants of conflict tasks. Of course, assumptions are assumptions and thus subject to debate. After all, this is the purpose of this present comment.

Conceptually, DMC is based on the assumed time-course of both channels’ activation and the characteristic part is the pulse-like function used to model the activation in the automatic channel. RDMC is based on a change of the relative contribution of both channels due to an attentional shift that happens over time. Albeit both models being embedded within dual-route frameworks, we believe that the underlying assumptions, and thus the architectural design, are sufficiently different that RDMC is a separate type of model, and not merely a "revised DMC" (Lee & Sewell, 2024, Abstract).

That said, we acknowledge that RDMC fits data from Simon and flanker tasks well. With recent methodological advances it was possible to compare both models directly using information criteria. Although both models fit conflict task data qualitatively more or less comparable (especially after considering trial-by-trial variability in the starting point for DMC), RDMC has an advantage over DMC in terms of the plain log-likelihood and AIC values. Yet, when correcting for the number of parameters with respect to the BIC statistic, both models are comparable. Of course, this should not be over-interpreted as sufficient grounds to reject either RDMC or DMC (see Roberts & Pashler, 2000), especially since a model fit depends on the specific data at hand and the width of the search space (which may be tweaked both in favor of RDMC and DMC). However, the measurement properties in terms of parameter recovery seem more problematic to us. Indeed, we come to a different conclusion than Lee and Sewell (2024). It seems that several parameters central to RDMC are not recovered reliably when the optimization algorithm is not provided with close-to-optimal starting values. We believe that further investigation is required to clarify the underlying problems.

Although our critical stance can obviously not be concealed, we welcome and much appreciate the development of RDMC (Lee & Sewell, 2024). Coming up with a model (architecture) able to predict different types of delta functions (i.e., negatively- vs. positively-sloped ones) has been a difficult task in the past. In fact, only recently, models have been put forward that are capable of predicting negatively-sloped delta functions (among them are DMC and RDMC, but see also Heuer et al., 2023; Hübner & Töbel, 2019; López & Pomi, 2024; Miller & Schwarz, 2021). So we acknowledge the advancement that RDMC provides in this matter, and are certain that it is an important contribution to the literature. There are also effects that RDMC can handle, but DMC cannot without additional assumptions. For example, DMC cannot account for reversed congruency effects without additionally assuming an undershoot of the pulse-function (see Koob, Mackenzie et al., 2023).

To this end, it would be desirable to see whether a more parsimonious version of the RDMC architecture can be developed (in particular, one avoiding the different parameters $k_c$ and $k_i$ for congruent and incongruent trials). If this were possible, it will further be interesting to see whether this model and DMC make different predictions that can be used to experimentally distinguish them. At present, though, and for the reasons discussed in this article, we do not see an advantage of RDMC over DMC. This might change in the future, of course, and we hope to stimulate a discussion and future research with this article.

## Supplementary Information

Below is the link to the electronic supplementary material.

Supplementary file 1 (pdf 460 KB)

## Data Availability

Not Applicable.

## Footnotes

1 Note that we use $\nu$ and $\mu$ to denote drift rates in the context of RDMC and DMC, respectively, for consistency with the original publications.

2 This reasoning might be underlined when thinking of the evidence accumulation process as a difference between two response counters $X_1$ and $X_2$ (i.e., $D = X_1 - X_2$). If the task-irrelevant feature provides evidence for $X_1$, as in congruent trials, *D* will be positive. A reduction of *D* may then be achieved by two means, either by (a) actively sampling evidence for $X_2$ or (b) reducing the counter of $X_1$. We believe that DMC’s architecture builds upon the latter mechanism.

3 Of course, alternative and non-inhibitory accounts for the mentioned phenomena are conceivable and have been proposed as well. We do not want to neglect this possibility, but rather provide examples to underscore our point that inhibition can be conceived as being able to reduce activation. After all, however, we also believe that passive decay of activation makes sense as well.

4 Allowing parameters to vary between congruent and incongruent trials has also been discussed for DMC’s amplitude *A* by Ulrich et al. (2015) and been implemented by Evans and Servant (2022) (to distinguish facilitation and interference in congruency effects). A critical difference, however, is that RDMC requires congruency-dependent parameters to predict negatively-sloped delta functions, while DMC does not.

5 A further complication might arise when flanker and target are presented with a delay and not simultaneously (e.g., Mackenzie et al., 2022; Schumacher et al., 2011). We won’t discuss this situation further, though, as the original version of DMC was technically developed for situations with simultaneous onsets.

6 The data reported in Ulrich et al. (2015) and used by Lee and Sewell (2024) and later in the present study as well were obtained from a Simon task with letters presented to the left or right, while the flanker task was one with two stimuli mapped to two responses. At least for the just mentioned variant of Simon task, for example, a letter does not hold intrinsic left or right information. Thus, we believe, some target information must be extracted in such cases.

7 Note that fitting DMC without a variable starting point, when indeed such a variability is present in the data, might distort the parameters of the model.

8 As was described above, this can be expected, as DMC typically requires a variable starting point to better account for fast errors. When we re-ran our analyses with a variable starting point for DMC (see Electronic Supplement C), qualitative model fits were more similar.

9 Note that our conclusions about the poor parameter recovery do not depend on the cost function nor on the specific minimization routine. When preparing the present manuscript, we explored various combinations of both the cost function (i.e., $\chi ^2$ vs. log-likelihood) and minimization routine (i.e., Nelder-Mead vs. differential evolution). In the end, however, parameter recovery for some of RDMC’s parameters was poor once we considered a larger parameter space during the estimation procedure. We do not want to deem RDMC as unreliable based on a single study, but at least we are skeptical about the very high correlations provided in the original study.

## Code availability

Code is available at https://osf.io/hn4uv/.
