# Supplementary material for: A comment on the Revised Diffusion Model for Conflict tasks (RDMC)
Source: Psychon Bull Rev. 2024 Oct 24;32(2):690–704. doi: 10.3758/s13423-024-02574-5 (PMC12000171; doi:10.3758/s13423-024-02574-5)
Supplement: Supplementary file 1 — (pdf 460 KB) [file 13423_2024_2574_MOESM1_ESM.pdf]

Electronic Supplement A: Mathematical Details of the Models

The Diffusion Model for Conflict tasks (DMC; Ulrich et al., 2015)

DMC translates dual-route models by describing the expected activation for one or the other response in the controlled and the automatic channel and the respective separate diffusion processes (see Fig. 1, left panel). The activation gathered within the controlled channel is linearly increasing with time t, similar to standard diffusion models (e.g., Ratcliff, 1978). Thus, the expected time-course of evidence accumulation via the controlled route is a straight line with $E[\mathbf{X_c}(t)] = \mu_c \cdot t$ (the straight black line in Fig, 1).

The expected time-course of the automatic channel $E[\mathbf{X_a}(t)]$ follows a pulse-like function that first increases and then decreases until reaching 0 again. Importantly, this activation is in the same or the opposite direction as that of the controlled channel in congruent and incongruent trials, respectively, and the evidence added in each time-step changes over time. Technically, a rescaled Gamma function is used to describe the expected time-course of activation within the automatic channel, that is, a Gamma function multiplied with A to determine the amplitude (see the dotted green and red lines in Fig. 1, left panel):

$$E[\mathbf{X_a}(t)] = A \cdot e^{-t/\tau} \cdot \left[\frac{t \cdot e}{(a-1) \cdot \tau}\right]^{a-1} .$$

Note that with $a = 2$ the maximum amplitude A is reached at $t = \tau$. A is positive-signed for congruent trials and negative-signed for incongruent trials.

Superimposing both diffusion processes by adding them yields the expected overall time-courses of activation in congruent and incongruent trials (see the green and red lines in Fig. 1, left panel):

$$E[\mathbf{X}(t)] = E[\mathbf{X_c}(t)] + E[\mathbf{X_a}(t)] .$$

These assumed expected time-courses of the activations within the controlled and the automatic channel are the essential parts of DMC. The respective drift rates are the first

derivatives with respect to time t (see Cox & Miller, 1965; Schwarz, 2022), hence

$$\mu_c(t) = \frac{dE[\mathbf{X_c}(t)]}{dt} = \mu_c$$

and

$$\mu_a(t) = \frac{dE[\mathbf{X_a}(t)]}{dt} = A \cdot e^{-t/\tau} \cdot \left[\frac{t \cdot e}{(a-1) \cdot \tau}\right]^{a-1} \cdot \left[\frac{(a-1)}{t} - \frac{1}{\tau}\right].$$

The overall time-dependent drift rate is then given by

$$\mu(t) = \frac{dE[\mathbf{X}(t)]}{dt} = \mu_c + \mu_a(t)$$

which varies between congruent and incongruent trials because of the different sign of A in $\mu_a(t)$.

Because the expected activation in the automatic channel decreases after reaching its maximum at time $t = (a-1) \cdot \tau$, the drift rate changes sign at this time-point.

The Revised Diffusion Model for Conflict tasks (RDMC; Lee & Sewell, 2024)

RDMC also builds on dual-route frameworks. In contrast to DMC, however, the basic idea is that both the controlled and the automatic channel continuously provide evidence, but their relative influence changes over time. At the beginning, the contribution of the automatic channel is large. Then, it decreases and at the same time, the contribution of the controlled channel increases reciprocally.

An exponential decay function

$$w_a(t) = A_0 e^{-kt}$$

models the decreasing contribution of the automatic channel where $A_0 \in [0; 1]$ represents the initial contribution of the automatic channel at time $t = 0$ and k the decay rate. (Note that this does not mean that any activation already exists at $t = 0$.) The contribution of

the controlled channel is based on $w_a(t)$ as

$$w_c(t) = 1 - w_a(t)\,.$$

Importantly, RDMC assumes separate decay rates for congruent and incongruent trials, that is, k_c and k_i (see the first and second row of Fig. A1 with $k_c \in \{2, 20\}$ and $k_i = 30$ in the left and right column).

Multiplication of these weights with the base drift rate of each channel yields the time-dependent drift rates

$$v_a(t) = w_a(t)d_a \quad \text{and} \quad v_c(t) = w_c(t)d_c$$

of the two channels. While the base drift rate for the controlled channel, d_c, is always positive, the base drift rate for the automatic channel, d_a, is positive on congruent and negative on incongruent trials and hence the overall drift rate $v(t)$ is

$$v(t) = \begin{cases} v_c(t) + v_a(t) = w_c(t)d_c + w_a(t)d_a & \text{for congruent trials} \\ v_c(t) - v_a(t) = w_c(t)d_c - w_a(t)d_a & \text{for incongruent trials.} \end{cases}$$

Further expanding these equations out yields

$$v(t) = \begin{cases} [(1 - A_0 e^{-k_c t})d_c] + (A_0 e^{-k_c t} d_a) & \text{for congruent trials} \\ [(1 - A_0 e^{-k_i t})d_c] - (A_0 e^{-k_i t} d_a) & \text{for incongruent trials} \end{cases}$$

as the time-dependent drift rates for both trial types. The last row of Figure A1 visualizes the expected values based on $v_c(t)$, $v_a(t)$, and $v(t)$ separately for congruent and incongruent trials and different values for k_c in the left and right column.

Figure A1

Illustration of RDMC.

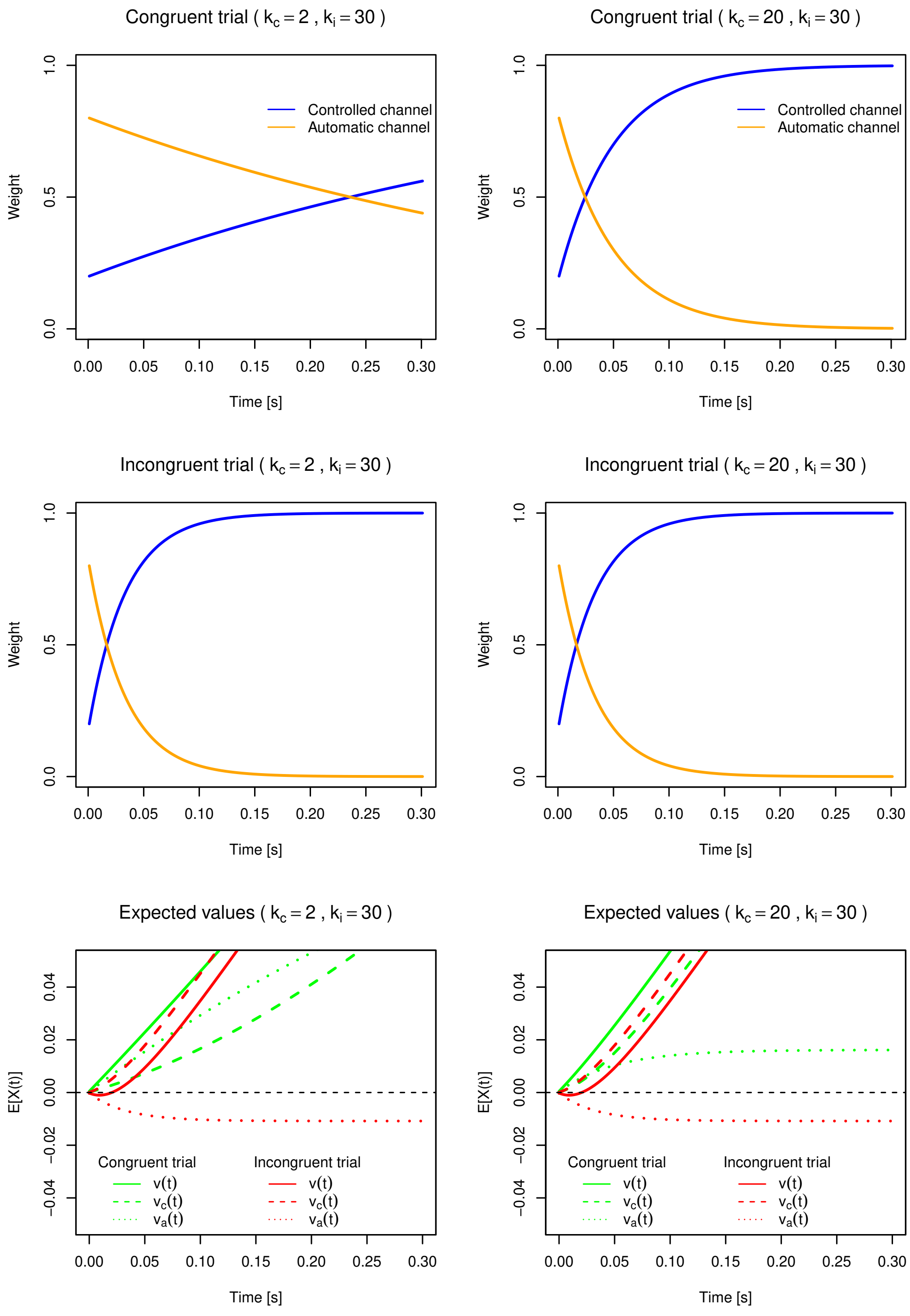

Note. The first two rows illustrate the weight function for the controlled and the automatic channel for congruent and incongruent trials. The last row visualizes the expected values based on $v_c(t)$, $v_a(t)$, and $v(t)$ separately for congruent (green) and incongruent (red) trials. For both columns we set $k_i = 30$ while $k_c = 2$ and $k_c = 20$ were used in the left and the right column, respectively.

Electronic Supplement B: Details on Parameter Ranges and the Parameter Recovery

When conducting our parameter recovery, we considered two parameter spaces. The first parameter space defines the range of uniform distributions from which the sets of original parameters were sampled for the synthetic data. The second parameter space constrained the search space when estimating the parameters via Differential Evolution. Naturally, the search space was set slightly larger than the space for drawing the original parameter values. Ranges for both parameter spaces are shown in Table B1.

The ranges for A_0, k_c, k_i, d_a, d_c, and b for the original parameters were as in Lee and Sewell (2024, Table 2). However, two things have to be mentioned to avoid confusion. First, we approximated the models' probability density function in the unit of seconds and with a diffusion constant of $\sigma = 1$. Lee and Sewell simulated RDMC also in the unit of seconds, but used a diffusion constant of $\sigma = 0.1$. Thus, to ensure equivalence, d_a and d_c in Table B1 need to be divided by 10. In addition, Lee and Sewell quantified the decision boundaries of RDMC in terms of the boundary separation, which is the distance between boundaries. In the present study, we assumed that b is the upper decision boundary (indicating a correct response), and $-b$ the lower decision boundary (indicating an incorrect response; see also Ulrich et al., 2015). Thus, b in Table B1 needs to be divided by 10 and multiplied by 2 to match the ranges of the boundary separation used by Lee and Sewell. The parameters A_0, k_c, and k_i are independent of the scaling of the evidence space, and thus do not require transformation.

For DMC, the parameter ranges for μ_c, A, and τ from which the original parameters were sampled were based on typical parameter ranges for DMC (see, e.g., Mackenzie & Dudschig, 2021; Ulrich et al., 2015). Readers familiar with the original paper on DMC (or the R-package DMCfun; Mackenzie & Dudschig, 2021) might be more accustomed to parameter values in the unit of milliseconds and with a diffusion constant of $\sigma = 4$. To convert the parameter values of the present study to units more typical in the context of

DMC, we can use the following set of equations (see also Koob, Mackenzie, et al., 2023, Appendix B):

$$\mu_{ms} = \frac{4 \cdot \mu_s \cdot \sqrt{1000}}{1000}$$
$$A_{ms} = 4 \cdot A_s \cdot \sqrt{1000}$$
$$\tau_{ms} = \tau_s \cdot 1000$$

Thus, for instance, a value of $\mu_c = 4$ in the present study refers to $(4 \cdot 4 \cdot \sqrt{1000})/1000 = 0.51$ in the unit of milliseconds and with a diffusion constant of $\sigma = 4$. Note that the equation for A can also be used for transforming b.

Table B1

Parameter Spaces for Drawing the Original Parameter Sets and When Estimating the Parameters

	Models/Parameters										
	RDMC					DMC			Both		
Space For..	A_0	k_c	k_i	d_a	d_c	μ_c	A	τ	b	t_0	s_{t0}
Original Parameters											
Lower	0.60	0	20	3.0	4.0	2.0	0.050	0.025	0.35	0.275	
Upper	0.90	80	80	7.0	8.0	6.0	0.200	0.150	0.75	0.400	
Estimating Parameters											
Lower	0.55	0	15	2.5	3.5	1.5	0.025	0.015	0.30	0.250	0.05
Upper	0.95	85	85	7.5	8.5	6.5	0.225	0.160	0.80	0.425	0.15

Figures B1 and B2 visualize scatter plots of recovered/estimated versus original parameter values. The closer the dots are to the diagonal line, the better is the recovery property. Qualitatively, we see that DMC has reasonable parameter recovery. Parameters μ_c, b, and t_0 are always recovered well, even for moderate trial numbers. The parameters associated with the automatic channel (i.e., A and τ) show acceptable parameter recovery for smaller trial numbers, and good recovery for high trial numbers. For RDMC, parameter recovery of d_c, b, and t_0 was always reasonable/good, even for small trial numbers. For A_0,

k_c, k_i, and d_a, the recovery was not ideal for small trial numbers. However, recovery became better for k_i and d_a with larger trial numbers, although really high trial numbers of 10,000 trials were necessary to achieve good parameter recovery. Finally, A_0 and k_c were always recovered rather poorly (see Table 2 in the main text).

Figure B1

Parameter Recovery for DMC

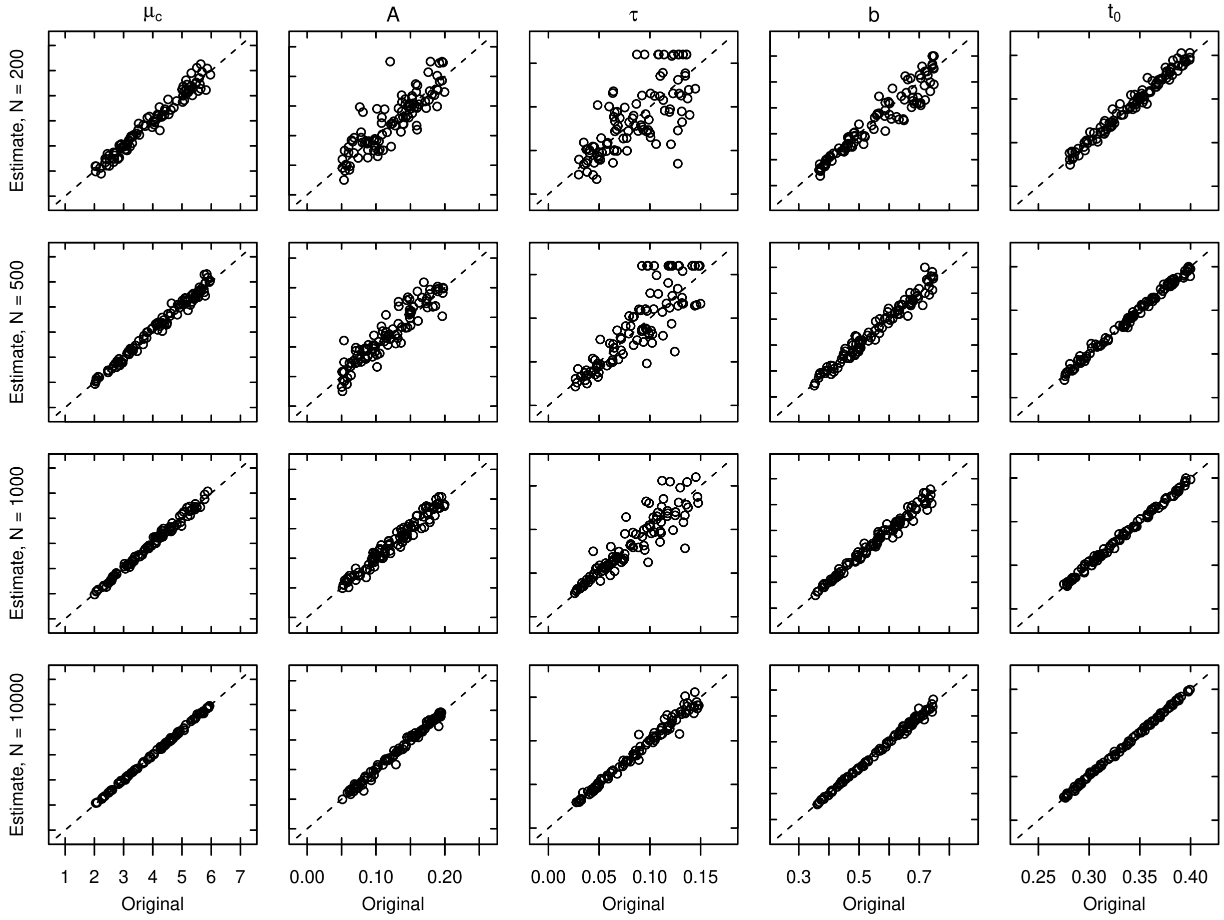

Note. Each panel shows recovered/estimated (y-axis) versus original (x-axis) parameters. Each parameter is presented in one column (see the letter at the top). Each trial number is presented in one row (see the axis labels at the leftmost y-axis). The closer the dots are to the diagonal line, the better is the parameter recovery.

Figure B2

Parameter Recovery for RDMC

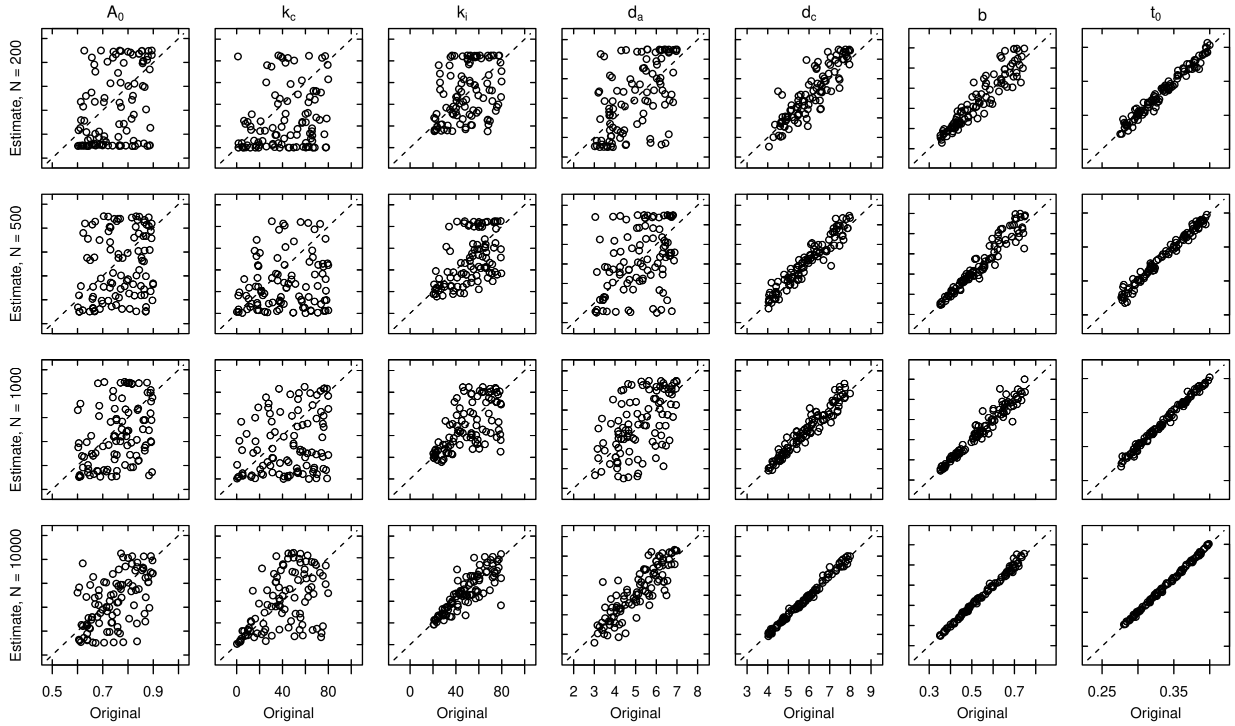

Note. Figure legend is identical to Figure B1

Electronic Supplement C: Model Fit and Comparison With Variability in the Starting Point for DMC

In the main text, we fitted DMC without a variable starting point. Oftentimes, however, variability in the starting point is necessary for DMC to account for fast errors reasonably well (Ulrich et al., 2015). We here provide qualitative model fits and the results from our model comparison, when including variability in the starting point for DMC. As in previous publications on DMC, variability in the starting point was realized with a symmetric Beta function, with shape and rate parameter α, scaled between b and $-b$. The lower and upper boundaries for α when estimating the model were set to 3 and 8, respectively. The qualitative model fits for DMC and RDMC with respect to the Simon and flanker data of Ulrich et al. (2015) are shown in Figures C1 and C2, respectively. As is evident, both models fitted the data equally well.

Table C1 further summarizes the results when re-running our model comparison. In short, RDMC slightly outperformed DMC with respect to the plain log-likelihood and the AIC statistic. When conservatively considering the number of parameters via the BIC statistic, both models were comparable.

Figure C1

DMC (upper row) and RDMC (lower row) Predictions for the Simon Task Data

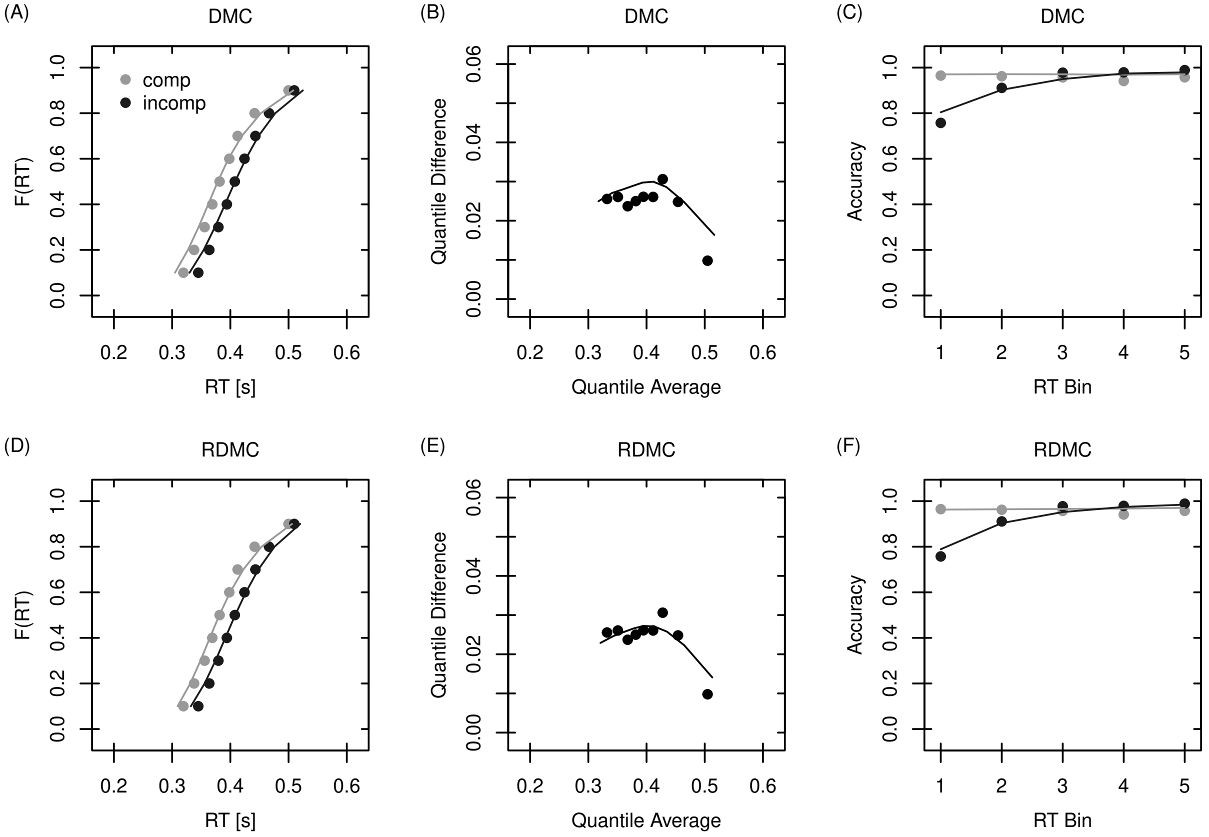

Note. In all panels, model predictions are shown as lines, and observed values are shown as dots. Specific values were obtained by averaging across participants. The upper and lower row show model predictions for DMC and RDMC, respectively. The leftmost panels (i.e,. A and D) show predicted and observed quantiles. The middle panels (i.e., B and E) show delta functions which can be derived by plotting the difference between quantiles (to the same probability) of incompatible and compatible trials against their mean. The rightmost panels (i.e., C and F) show CAFs, derived by first binning RTs and then calculating the proportion of correct responses per bin. For RDMC, this figure visualizes the same information as Figure 2 in the main text.

Figure C2

DMC (upper row) and RDMC (lower row) Predictions for the Flanker Task Data

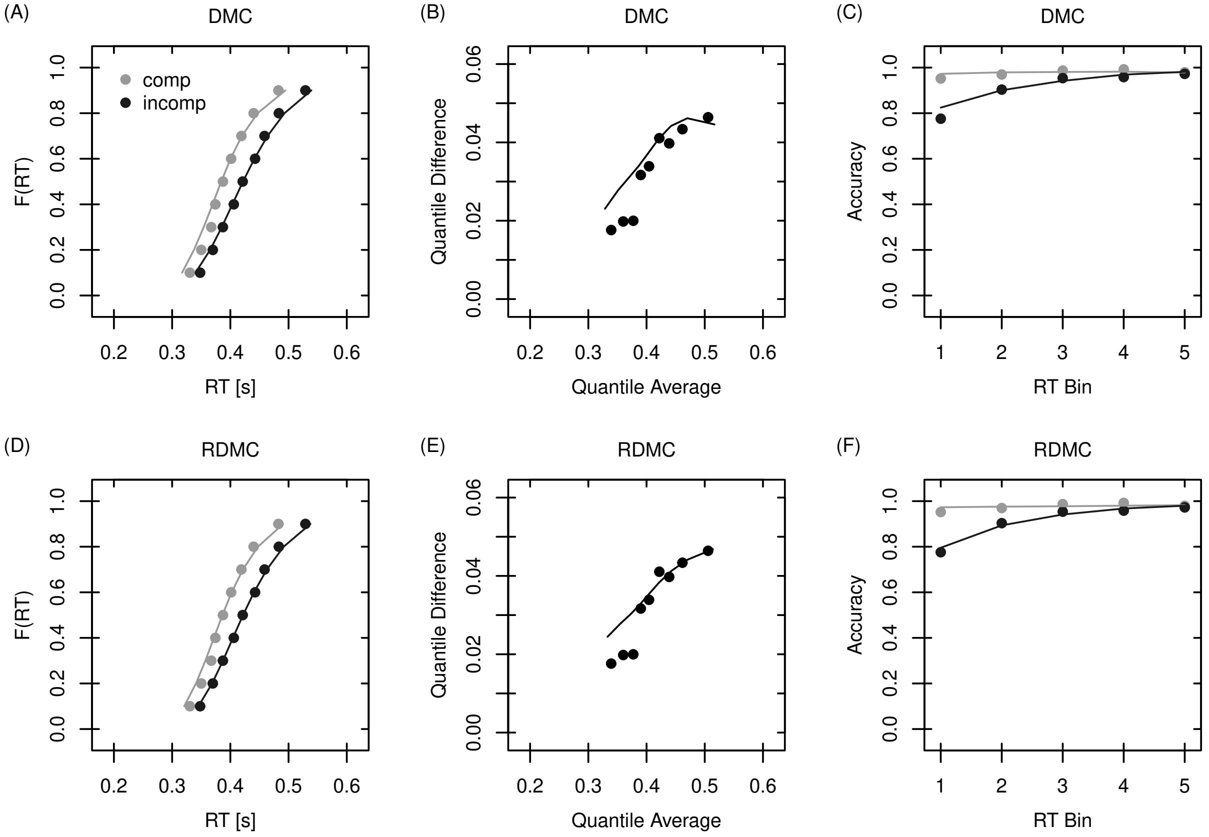

Note. Figure legend is identical to Figure C1. For RDMC, this figure visualizes the same information as Figure 3 in the main text.

Table C1

Summary of Log-Likelihood, AIC, and BIC Values for DMC and RDMC, Separately for the Simon and Flanker Data Sets

	Log-Likelihood		AIC		BIC	
Dataset/Model	*M*	*N*	*M*	*N*	*M*	*N*
Simon						
DMC	356	4	-697	5	-671	8
RDMC	359	12	-702	11	-672	8
Flanker						
DMC	373	5	-732	7	-705	10
RDMC	375	11	-734	9	-704	6

Note. AIC = Akaike information criterion, BIC = Bayesian information criterion, *M* = Mean, *N* = Number of participants for which a certain statistic was largest (log-likelihood) or smallest (AIC and BIC; i.e., for which a certain model turned out 'best').
